# 

**Published:** 1881-05

**Authors:** 


### CONTENTS:

PAGE.

ORIGINAL COMMUNICATIONS:

Recent Researches on the Action of Cholagogues. By H. S. Kilbourne, M. D., - 433
Albumen in the Urine. By Chas. A. Doremus, M. D., . . . . . . . 440

CLINICAL REPORTS:

A Case of Extra-Uterine Pregnancy.—Tapping of Sac.—Recovery. By James P. White. Reported by G. H. Van Deusen, M. D., . . . . . . . . 446

TRANSLATIONS:

On the Therapeutical Value of the Injection of Medicated Fluids through the Eutachian Tube.—Extracts from a paper by Prof. Jos. Gruber. From the German by Dr. Lucien Howe, . . . . . 449

SELECTIONS:

The Catgut Ligature, . . . . . . 453
Further Researches on Ethylate of Sodium in the Treatment of Nævus and other forms of disease. By Benjamin Ward Richardson, M. D., F. R. S., . . . 459
Uterine Disease. By Edward C. Mann, M. D., . . . . . . . . . 463
A Simple Method for Improving the Odor of Iodoform. By Dr. J. Hanlo, . . . 464
Adhesion of the Placenta. By A. Cummins Air, L. R. C. P., . . . . . . 465
On Hyoscyamin in Mental Diseases—Seppilli and Riva, . . . . . . . 467
Artificial Respiration in New-born Children, 468
What Shall we do with Inebriates? By Dr. Crother, . . . . . . . . . 470
Enteric Fever, . . . . . . . 471

SOCIETY REPORTS:

Buffalo Medical Association, . . . . 472

EDITORIAL:

The Sale of Poisonous Drugs, . . . . 475
The Buffalo Medical College—Resignation of Prof. E. V. Stoddard, . . . . . 477

REVIEWS, . . . . . . . . 478

